# Supplementary material for: Isolation and Characterization of Erianthus arundinaceus Phosphate Transporter 1 (PHT1) Gene Promoter and 5′ Deletion Analysis of Transcriptional Regulation Regions under Phosphate Stress in Transgenic Tobacco
Source: Plants (Basel). 2023 Nov 3;12(21):3760. doi: 10.3390/plants12213760 (PMC10650210; doi:10.3390/plants12213760)
Supplement: Supplementary file 1 [file plants-12-03760-s001.zip › plants-2669401-supplementary.pdf]

Supplementary file

A

ATGGCGCGCGGGGGCGACGGCCTGCAGGTGCTCAGCGCGCTGGACGCGGCCAAGACGCAGTGGTACCACTTCACG
GCCATCATCGTGGCCGGCATGGGCTTCTTCACGGACGCCTACGACCTCTTCTGCATCTCCCTCGTCACCAAGCTGCTGG
GCCGCATCTACTACACGGACACCAGCAAGGACAACCCGGGCTCGCTCCCGCCCAACGTCGCCGCGGCGGTCAACGG
CGTCGCCTTCTGCGGCACGCTCGCCGGCCAGCTCTTCTTCGGCTGGCTCGGCGACAAGCTCGGGCGCAAGAGCGTG
TACGGGATGACGCTCATGCTCATGGTCATCTGCTCCGTCGCGTCGGGCCTCTCCTTCGGCCACACCCCCACGGGTGTC
ATGGCCACGCTCTGCTTCTTCCGCTTCTGGCTCGGGTTCGGCATCGGCGGGGACTACCCGCTGTCCGCCACCATCATG
TCACGAGTACGCCAACAAGAAGACCCGCGGCGCCTTCATCGCCGCCGTCTTCGCCATGCAGGGCTTCGGCATCCTCG
CCGGTGGCATTGTCACGCTCATCATCTCCGCCGCCTTCCGCGCGGGGTACCCGGCCCCGGCGTACAGGGACGACCAC
TTCAACTCCACCGTGCCGCAGTCCGACTTCGTGTGGCGCATCATCCTCATGCTCGGCGCCTTGCCGGCGGTGCTCACC
TACTACTGGCGGATGAAGATGCCCGAGACGGCGCGCTACACGGCGCTGGTGGCCAAGAACGCCAAGCAGGCCGCG
GCCGACATGTCCAAGGTGCTCCAGACGGAGATCGTCGACGAGCAGGAGAAGCTGGACCAGATGGTCACCGCCGAG
AGCAACACCTTCCGGCCTCTTCTCCAGTGAGTTCGCGCGCCGCCACGGCCTCCACCTCGTCGGCACCGCCAACCACG
TGGTTCCTGCTCGACATCGCCTTCTACAGCCAGAACCTGTTCCAGAAGGACATCTTCACAGCCATCAACTGGATCCCCA
AGGCCAACACCATGAGCGCGCTCGAGGAGGTGTTCCGCATCTCCCGCGCCCAGACGCTCATCGCGCTCTGCGGCACC
GTCCCGGGGTACTGGTTCACCGTCGCGCTCATCGACGTCGTCGGCCGCTTCGCCATCCAGCTGCTCGGATTCTTCATG
ATGACCGTCTTCATGCTCGGCCTCGCCATCCCCTACCACCACTGGACCACGAAAGGGAACCACATCGGATTCGTCGTC
ATGTACGCCTTCACCTTCTTCTTCGCGAACTTCGGGCCCAACAGCACCACCTTCATCGTGCCGGCCGAGATCTTCCCGG
CGCGGCTGCGGTCCACCTGCCACGGCATCTCCGCCGCCTCGGGGAAGGCCGGAGCCATCATCGGCGCCTTCGGGTT
CCTGTACGCGGCGCAGAACCAGGACAAGAGCAAGACGGACGCCGGGTACCCCGCGGGCATCGGCGTGCGCAACTC
GCTCTTCGTCCTCGCTGCCTCCAACATGCTCGGCTTCGTCCTCACCTTCCTCGTGCCGGAGTCCAAGGGCAAGTCGCT
CGAGGAGATGTCCGGTGAGGCTGACGACGCCGAGGAGGAGGCCGTCGGCACCCGCGCGGTGCGGCCGTCGGAGA
CCCAGATGGTGTAG

B

MARGGDGLQVLSALDAAKTQWYHFTAIIVAGMGFFTDAYDLFCISLVTKLLGRIYYTDTSKDNPGSLPPNVAAAVNGV
AFCGTLAGQLFFGWLGDKLGRKSVYGMTLMLMVICSVASGLSFGHTPTGVMATLCFFRFWLGFGIGGDYPLSATIMS
EVRQQEDPRRLHRRRLRHAGLRHPRRWHCHAHHLRRLPRGVPGPGVQGRPLQLHRAAVRLRVAHHPHARRLAGG
AHLLLADEDARDGALHGAGGQERQAGRGRHVQGAPDGDRRRAGEAGPDGHRREQHLPASSPVSSRAATASTSSAP
PTTWFLLDIAFYSQNLFQKDIFTAINWIPKANTMSALEEVFRISRAQTLIALCGTVPGYWFTVALIDVVGRFAIQLLGFF
MMTVFMLGLAIPYHHWTTKGNHIGFVVMYAFTFFFANFGPNSTTFIVPAEIFPARLRSTCHGISAASGKAGAIIGAFGF
LYAAQNQDKSKTDAGYPAGIGVRNSLFVLAASNMLGFVLTFLVPESKGKSLEEMSGEADDAEEEAVGTRAVRPSETQ
MV

Figure S1. The nucleotide sequence of *Erianthus arundinaceus* Phosphate transporter 1;2 (EaPHT1;2). (A) The full-length CDS sequence of the EaPHT1;2 gene; (B) The ExPasy-translated protein sequence of the EaPHT1;2.

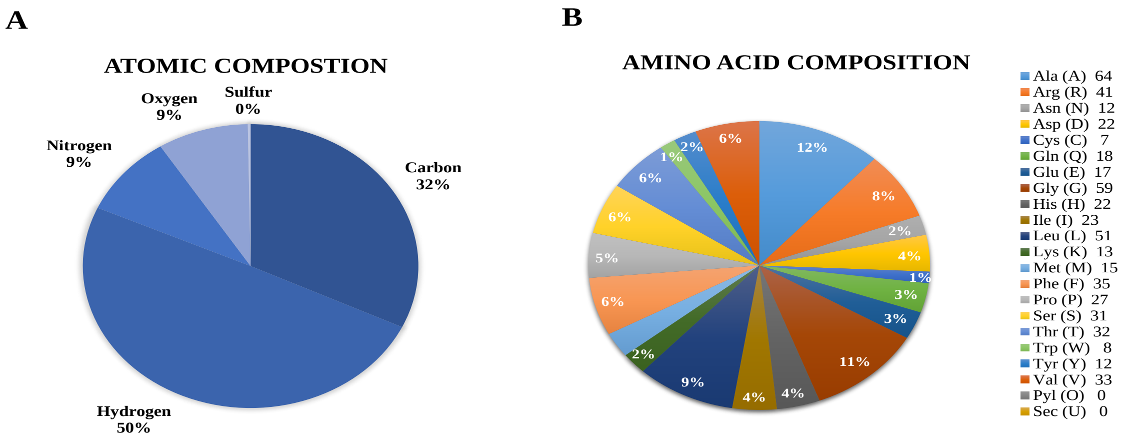

Figure S2: Physicochemical parameters of the selected protein: (A) Atomic composition of EaPHT1;2 protein, (B) Amino acid composition of EaPHT1;2 protein

A

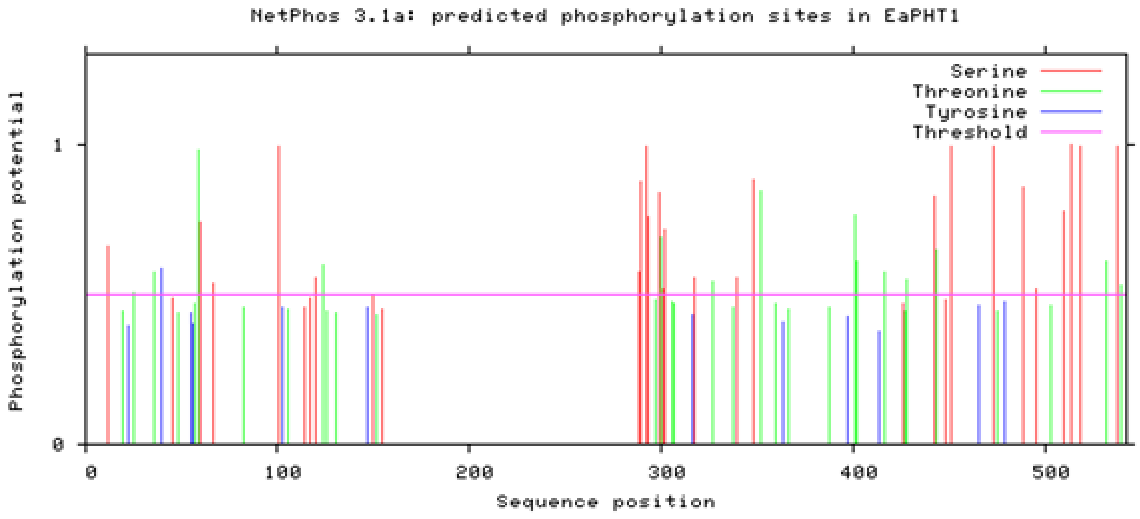

B

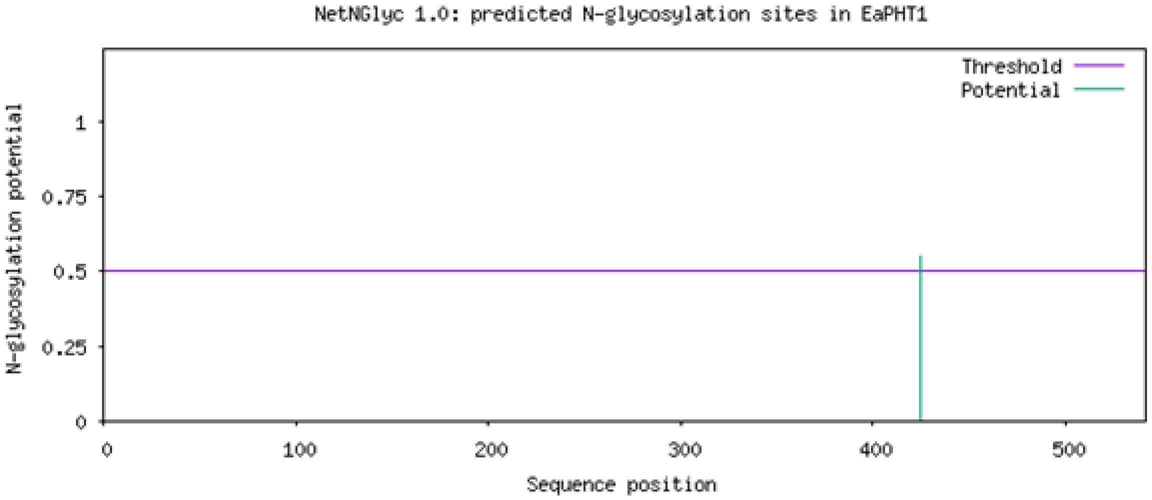

Figure S3: Post-translation modification sites of EaPHT1. (A) Phosphorylation sites. (B) Glycosylation site. The amino acids are represented in different lines at their position in the sequence and the scores higher than the threshold value are considered for posttranslational modification.

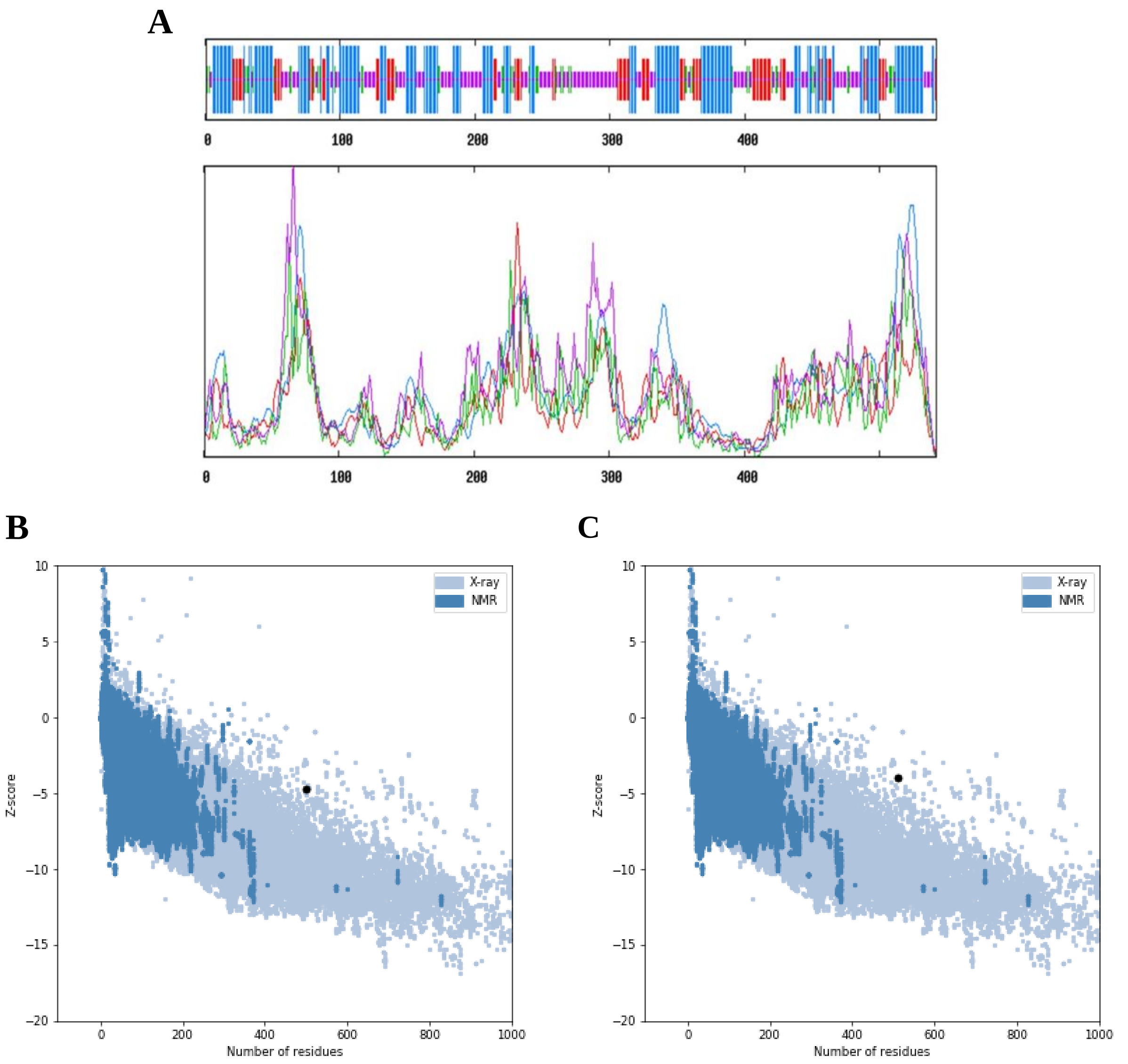

Figure S4: Protein structure prediction and evaluation. (A) Secondary elements in the EaPHT1 protein predicted using the SOPMA tool. (B) Structural evaluation of EaPHT1;2 obtained through Phyre2 model; (B) EaPHT1;2 obtained through Swiss-Modelling.

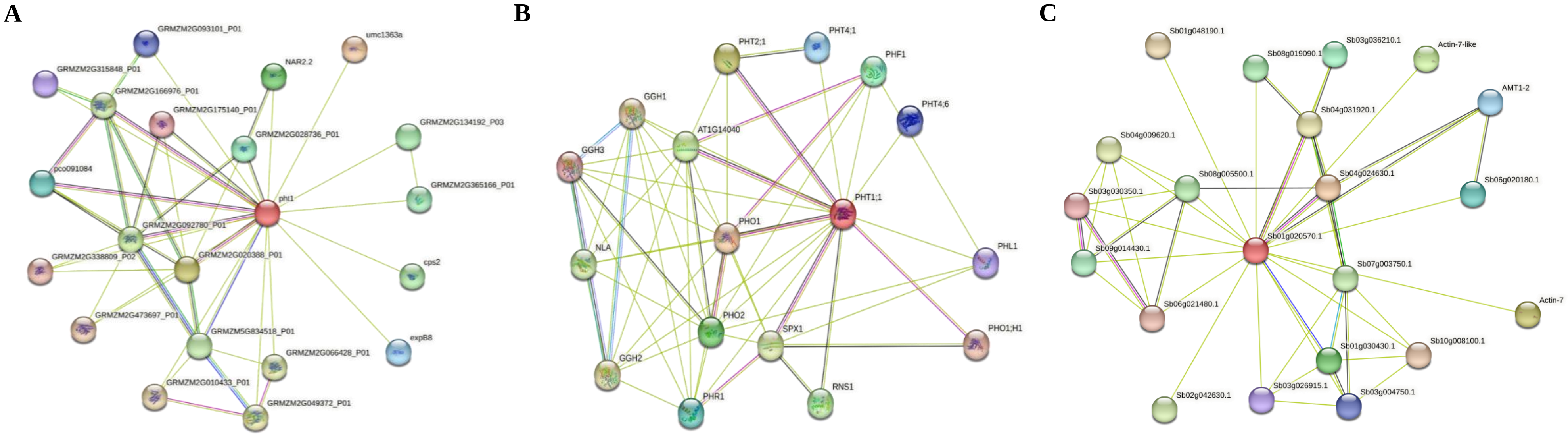

Figure S5: Protein-protein Interaction analysis of EaPHT1;2 protein. (A) PPI of EaPHT1;2 with ZmPHT1 protein. (B) PPI of EaPHT1;2 with AtPHT protein. (C) PPI of EaPHT1;2 with SbPHT protein.

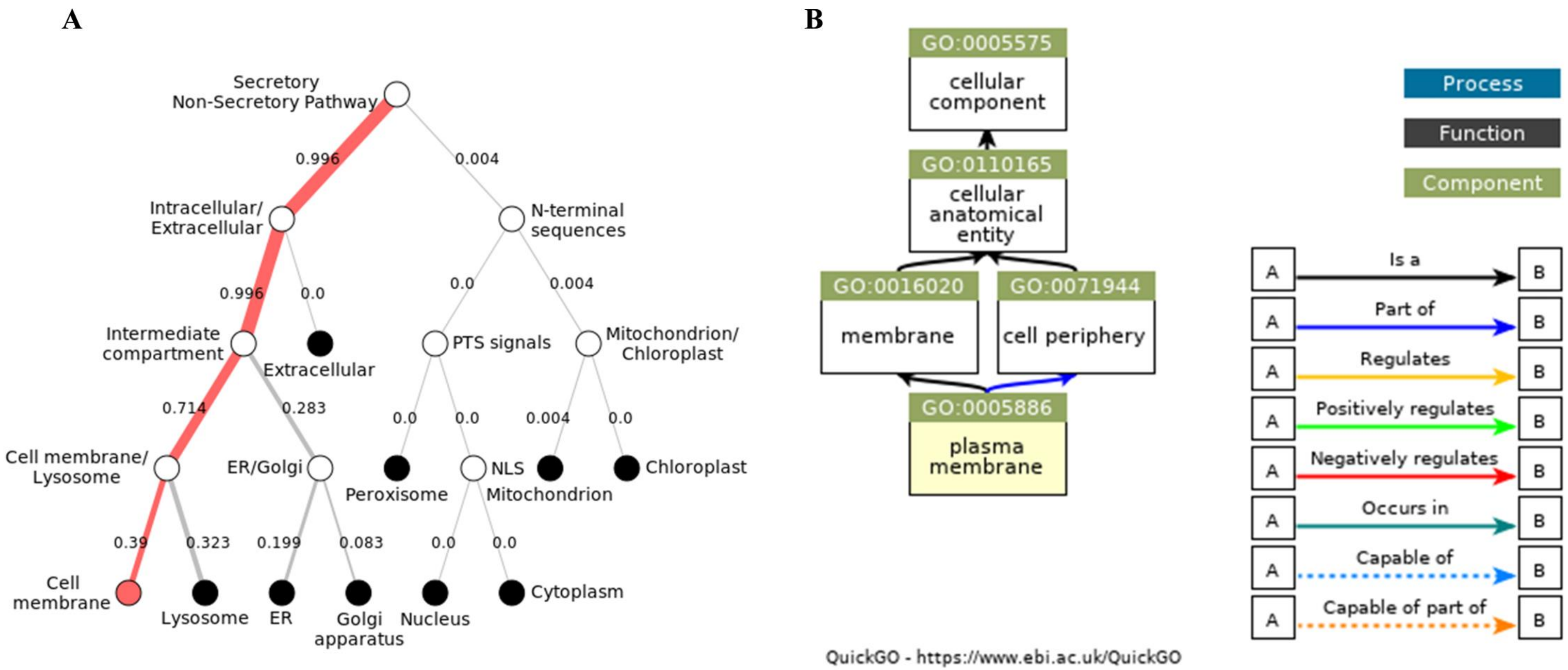

.

Figure S6: Subcellular Localization analysis (A) EaPHT1;2 protein localization using online tools DeepLoc analysis (B) QuickGo Term analysis of EaPHT1;2 protein

A

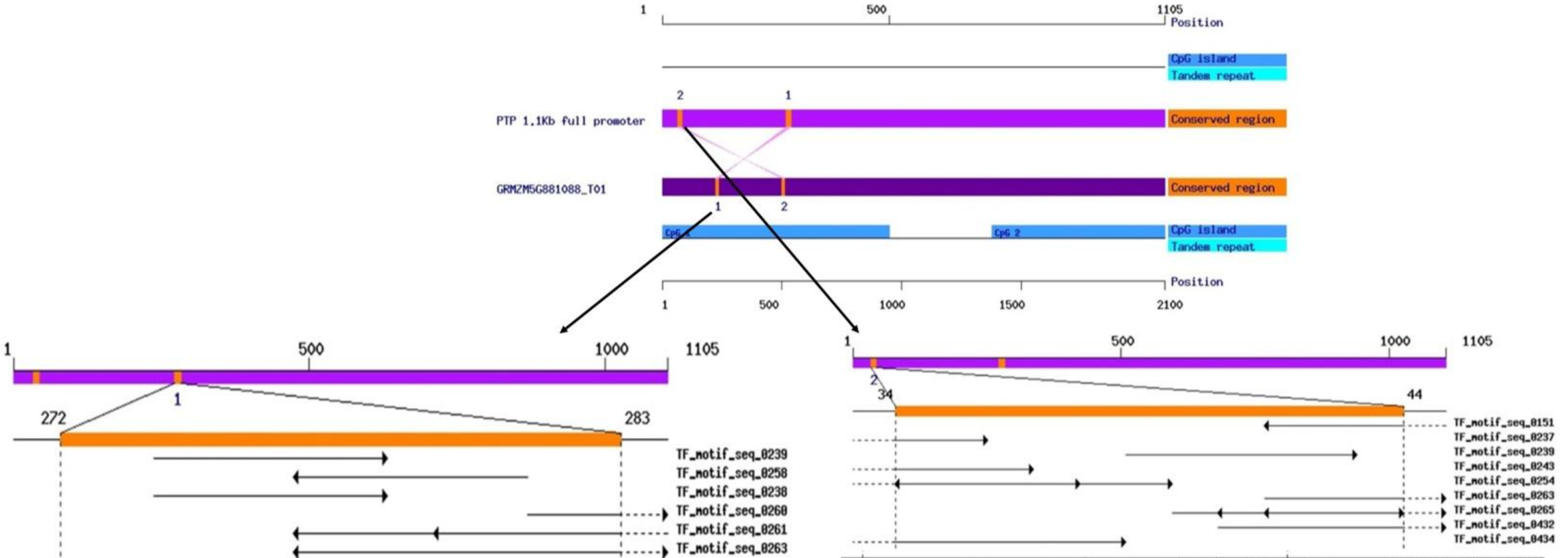

Matrix ID	TFs/TFBSs	Position	Strand	Similarity score	Hit sequence	Located in Tandem repeat	Located in CpG island
TF_motif_seq_0239 (./TFBSinfo.php? matrix=TF_motif_seq_0239)	AT1G29160, ...	274	+	1	AAAGT	NO	NO
TF_motif_seq_0258 (./TFBSinfo.php? matrix=TF_motif_seq_0258)	U01377	277	-	0.8	GTCGC	NO	NO
TF_motif_seq_0238 (./TFBSinfo.php? matrix=TF_motif_seq_0238)	DOFCOREZM	274	+	1	AAAGT	NO	NO
TF_motif_seq_0260 (./TFBSinfo.php? matrix=TF_motif_seq_0260)	OSE2ROOTNODULE	282	+	1	CTCTT	NO	NO
TF_motif_seq_0261 (./TFBSinfo.php? matrix=TF_motif_seq_0261)	SURECOREATSULTR11	277	-	0.8	GTCGC	NO	NO
		280	-	0.8	GCCTC		
TF_motif_seq_0263 (./TFBSinfo.php? matrix=TF_motif_seq_0263)	SORLIP1AT	277	-	0.8	GTCGC	NO	NO
		280	+	0.8	GCCTC		

Matrix ID	TFs/TFBSs	Position	Strand	Similarity score	Hit sequence	Located in Tandem repeat	Located in CpG island
TF_motif_seq_0151 (./TFBSinfo.php? matrix=TF_motif_seq_0151)	AT2G42400	42	-	0.75	gcccccttagAACGC	NO	NO
TF_motif_seq_0237 (./TFBSinfo.php? matrix=TF_motif_seq_0237)	AT1G51600, ...	31	+	1	GGATA	NO	NO
TF_motif_seq_0239 (./TFBSinfo.php? matrix=TF_motif_seq_0239)	AT1G29160, ...	39	+	1	AAGGC	NO	NO
TF_motif_seq_0243 (./TFBSinfo.php? matrix=TF_motif_seq_0243)	AT1G51600, ...	32	+	1	GATAT	NO	NO
TF_motif_seq_0254 (./TFBSinfo.php? matrix=TF_motif_seq_0254)	AT3G14230	33	+	0.8	ATATA	NO	NO
		34	-	0.8	TATAT		
		35	+	0.8	ATATA		
TF_motif_seq_0263 (./TFBSinfo.php? matrix=TF_motif_seq_0263)	SORLIP1AT	42	+	0.8	GCCCC	NO	NO
TF_motif_seq_0265 (./TFBSinfo.php? matrix=TF_motif_seq_0265)	SORLIP2AT	40	+	0.8	AGGCC	NO	NO
		41	+	0.8	GGCCC		
		41	-	1	GGCCC		
		42	-	0.8	GCCCC		
TF_motif_seq_0432 (./TFBSinfo.php? matrix=TF_motif_seq_0432)	GGTCCCATGMSAUR	41	+	0.75	GGCCCcct	NO	NO
TF_motif_seq_0434 (./TFBSinfo.php? matrix=TF_motif_seq_0434)	P1BS	31	+	0.83	GGATAtat	NO	NO

B

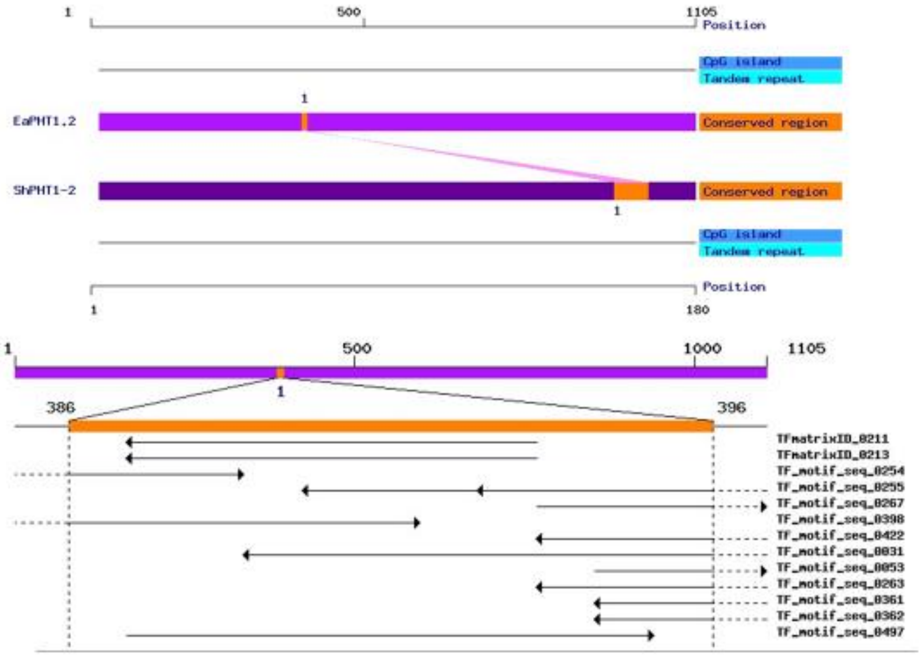

Matrix ID	TFs/TFBSs	Position	Strand	Similarity score	Hit sequence	Located in Tandem repeat	Located in CpG island
TFmatrixID_0211	Sb01g031920,...	387	-	1	tAGTGTt	NO	NO
TFmatrixID_0213	AC185108.3_FG011	387	-	1	tAGTGTt	NO	NO
TF_motif_seq_0254	AT3G14230	384	+	0.8	ATTTA	NO	NO
TF_motif_seq_0255	AT1G25560,...	390	-	1	TGTTG	NO	NO
		393	-	1	TGTTG		
TF_motif_seq_0267	AT5G01380	394	+	0.8	GTTGC	NO	NO
TF_motif_seq_0398	GRMZM2G136887_T02	385	+	0.86	TTTAGtg	NO	NO
TF_motif_seq_0422	U22102	394	-	0.75	gttGCATG	NO	NO
TF_motif_seq_0031	GCAACREPEATZMZEIN	389	-	0.7	gtgttGTTGC	NO	NO
TF_motif_seq_0053	SORLREP5AT	395	+	1	TTGCAtgact	NO	NO
TF_motif_seq_0263	SORLIP1AT	394	-	0.8	GTTGC	NO	NO
TF_motif_seq_0361	RYREPEATGMGY2	395	-	0.86	ttGCATG	NO	NO
TF_motif_seq_0362	RYREPEATLEGUMINBOX	395	-	0.96	ttGCATG	NO	NO
TF_motif_seq_0497	D4GMAUX28	387	+	0.89	TAGTGttgt	NO	NO

Figure S7: Comparative In-silico analysis of EaPHT1;2 promoter. (A) The EaPHT1;2 promoter conserved regions awith ZmPHT1 and (B) One conserved region was ShPHT1;2 promoter regions.

Table S1: List of primer sequences used in this study for EaPHT1-2 gene isolation, promoter isolation, promoter deletion constructs, and analysis.

Primers	Name	Primer sequence
Gene Isolation Primer	GPHT1F	ATGGCGCGCGGCGGGGACGGCCTGCA
	GPHT1R	CTACACCATCTGGGTCCCCGACGGC
Primary PCR Primer	ASP I	GGATCCTAATACGACTCACTATAGGGC
	pPHT R1	TAGTAGATGCGGCCGAGCAGC
Secondary PCR Primer	ASP II	AATAGGGCTCGAGCGGC
	pPHT R2	CCCATGCCGGCGATGACGATG
Promoter-specific primers	pFL F	CGCAAGCTTAAGGCCCCCTTAGAACGCG
	pFL R	TAAACCATGGGGTCGCTGCACC
GUS-specific Primers	GUS F	GGAATGGTGATTACCGACG
	GUSR	ATACCTGTTCACCGACGACG
Deletion Primers (For all deletion Reverse primer is the same)	EaPHT-pD1 F	CGCAAGCTTAGTGTTGTTGCATGACTG
	EaPHT-pD2 F	CGCAAGCTTAGCATCTCCATCTAACCACCA
	EaPHT-pD3 F	CGCAAGCTTAAGGCCCCCTTAGAACGCG
	EaPHT-pD4 F	CGCAAGCTTAGTGTTGTTGCATGACTG
	EaPHT-pD5 F	CGCAAGCTTAGCATCTCCATCTAACCACCA
	pFL R	TAAACCATGGGGTCGCTGCACC

Table S2: Physicochemical characteristics of the EaPHT1;2 protein

**Properties**	**Value**
No. of amino acids	542
Molecular weight	58940.73
Theoretical pI	9.8
No. of negatively charged residues (Asp + Glu)	38
No. of positively charged residues (Arg + Lys)	55
Total number of atoms	8262
Extinction Coefficient (all pairs of Cys residues form cysteines)	62255
Extinction Coefficient (all Cys residues are reduced)	61880
Half-life (in vitro) (h)	30
Instability index (II)	43.1
Aliphatic index	82.71
Grand average of hydropathicity	0.019

Table S3: Subcellular localization of EaPHT1-2 gene in different locations

**Localization**	**Likelihood**
Cell membrane	0.3897
Lysosome/Vacuole	0.31
Endoplasmic reticulum	0.2102
Golgi apparatus	0.083
Mitochondrion	0.0063
Peroxisome	0.0005
Plastid	0.0002
Nucleus	0.0001
Extracellular	0
Cytoplasm	0
